# Fucoxanthin Attenuates Rifampin-Induced Cytochrome P450 3A4 (*CYP3A4*) and Multiple Drug Resistance 1 (*MDR1*) Gene Expression Through Pregnane X Receptor (PXR)-Mediated Pathways in Human Hepatoma HepG2 and Colon Adenocarcinoma LS174T Cells

**DOI:** 10.3390/md10010242

**Published:** 2012-01-23

**Authors:** Cheng-Ling Liu, Yun-Ping Lim, Miao-Lin Hu

**Affiliations:** 1 Department of Food Science and Biotechnology, National Chung Hsing University, Taichung 402, Taiwan; Email: g9643203@mail.nchu.edu.tw; 2 Department of Pharmacy, College of Pharmacy, China Medical University, Taichung 404, Taiwan; 3 Department of Emergency, Toxicology Center, China Medical University Hospital, Taichung 404, Taiwan

**Keywords:** fucoxanthin, PXR, CYP3A4, MDR1, drug resistance, rifampin

## Abstract

Pregnane X receptor (PXR) has been reported to regulate the expression of drug-metabolizing enzymes, such as the cytochrome P450 3A (CYP3A) family and transporters, such as multiple drug resistance 1 (MDR1). Fucoxanthin, the major carotenoid in brown sea algae, is a putative chemopreventive agent. In this study, we determined whether fucoxanthin could overcome drug resistance through attenuation of rifampin-induced *CYP3A4* and *MDR1* gene expression by PXR-mediated pathways in HepG2 hepatoma cells. We found that fucoxanthin (1–10 μM) significantly attenuated rifampin (20 μM)-induced CYP3A4, MDR1 mRNA and CYP3A4 protein expression at 24 h of incubation. Mechanistically, fucoxanthin strongly attenuated the PXR-mediated CYP3A4 promoter activity in HepG2 cells. In addition, fucoxanthin attenuated constitutive androstane receptor (CAR)- and rPXR-mediated CYP3A4 promoter activity in this cell line. Using the mammalian two-hybrid assay, we found that fucoxanthin significantly decreased the interaction between PXR and SRC-1, a PXR co-activator. Thus, fucoxanthin can decrease rifampin-induced CYP3A4 and MDR1 expression through attenuation of PXR-mediated CYP3A4 promoter activation and interaction between PXR and co-activator. These findings could lead to potentially important new therapeutic and dietary approaches to reduce the frequency of adverse drug reactions.

## 1. Introduction

Fucoxanthin, the major non-provitamin A carotenoid found in *Undaria Pinnatifida*, has been shown to have many biological functions, such as suppression of adipocyte differentiation [1], anti-mutagenicity [2], anti-inflammation [3], and anti-cancer effects. Studies have shown that fucoxanthin exhibits anti-proliferative potential by inducing cell cycle arrest at the G0/G1 phase and apoptosis of various cell lines, such as prostate cancer PC-3, DU 145, and LNCaP cells [4], leukemia HL-60 cells [5], colon cancer HT-29, Caco-2, and DLD-1 cells [6]. Fucoxanthin can be hydrolyzed into fucoxanthinol by lipase, cholesterol esterase, carboxylesterase in intestinal Caco-2 cells and in mice [7] then subsequently converted into amarouciaxanthin a, which is a major metabolite of fucoxanthin in the liver of mice [8]. Moreover, Hashimoto *et al*. reported that fucoxanthinol is detectable at 44.2 nmol/L in human plasma in subjects orally administered with kombu extract containing 31 mg fucoxanthin for 24 h [9].

A lot of evidence indicates that phytochemicals found in fruit and vegetables exhibit chemoprevention and anti-drug resistance potential. For example, curcumin [10], epigallocatechin-3-gallate (EGCG) [11] and resveratrol [12] and its analogues [13], are useful in overcoming drug resistance in cancer. In addition, some carotenoids such as lycopene, zeaxanthin, and capsanthin have been shown to decrease drug resistance in human MDR1-transfected mouse lymphoma cells and human breast cancer cells, indicating that carotenoids are possible resistance modifiers in cancer chemotherapy [14]. However, it is unclear whether fucoxanthin itself is capable of overcoming drug resistance.

The cytochrome P450 (CYP) families are superfamily of hemethiolate-containing proteins, which metabolize a number of endogenous substrates such as steroids, eicosanoids, and xenobiotics including various carcinogens, toxins, and therapeutic drugs. CYP3A4 makes up the largest portion of the CYP family, which is mainly distributed in the liver and intestine and has clinical importance because it metabolizes at least 50% of marketed pharmaceutical agents [15]. Both expression and activity of CYP3A4 are also greatly regulated by many drugs and dietary chemicals, such as rifampin (an antibiotic), carbamazepine (an anticonvulsant), glucocorticoids, and hyperforin (a major component of St. John’s wort) [16]. In addition, the *MDR1* gene encodes P-glycoprotein (P-gp), which is a multidrug transporter that has a major role in drug resistance [17]. MDR1 has been found to promote the efflux of a wide range of structurally and functionally diverse compounds from cells, which decrease their intracellular accumulations [18,19]. The effectiveness of chemotherapy is often limited by drug resistance, and much effort has been expended to determine an approach to overcome this resistance [20].

Human pregnane X receptor (PXR), a member of the nuclear receptors (NRs) superfamily encoded by *NR1I2*, has been shown to be involved in protecting tissues from potentially toxic, exogenous and endogenous compounds, in drug metabolism, bile acid transport, biotransformation and clearance of widely used anticancer drugs as well as modulating the inflammatory response mediated by exogenous toxins [21,22,23]. Being a ligand-activated transcription factor, the PXR pathway is also activated by a large number of prescription drugs designed to treat infection, cancer, convulsion, and hypertension, and thus it is believed to play an important role in drug metabolism/efflux and drug-drug interactions [24]. Indeed, PXR activation has been shown to have an anti-apoptotic role in colon cancer cells [25] and induces FGF19-dependent tumor aggressiveness in humans and mice [26], whereas PXR antagonist decreased cell proliferation in breast cancer [27] and interfered with cancer drug resistance [28]. The activation of PXR can stimulate the expression of many important drug metabolism enzymes (DMEs) and ATP binding cassette (ABC) efflux transporters, which are able to prevent the intracellular accumulation of drugs by increasing excretion or efflux mechanisms [17]. Thus, PXR functions as a xenobiotic sensor [29] to coordinate and regulate drug clearance in the liver and intestine via transcriptional regulation of xenobiotic-detoxifying enzymes and transporters, such as cytochrome P450 (CYP) and multiple drug resistance 1 (MDR1) [30]. 

In this study, we determined whether fucoxanthin may overcome drug resistance through attenuation of rifampin-induced CYP3A4 and MDR1 gene expression by PXR-mediated pathways in HepG2 hepatoma cells. Mechanistically, we employed luciferase reporter gene assays to determine whether fucoxanthin attenuates the PXR-mediated CYP3A4 promoter activity. In addition, we used the mammalian two-hybrid assay to confirm the interaction between PXR and SRC-1, a main PXR co-activator. Our results may lead to the development of important new therapeutic and dietary approaches to reduce the frequency of undesirable drug interactions. 

## 2. Results

### 2.1. Fucoxanthin Inhibits the Basal and Attenuated Rifampin-Induced CYP3A4 Enzyme Activity in HepG2 Cells

**Figure 1:**
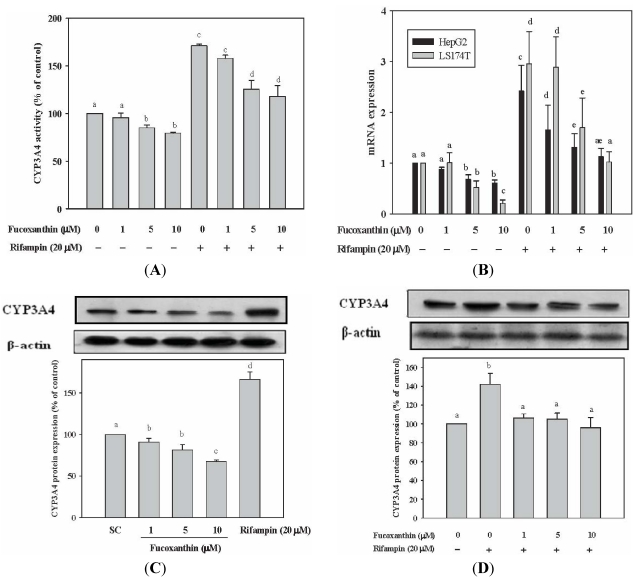
Effects of fucoxanthin (0–10 μM) alone or in combination with rifampin (20 μM) on CYP3A4 enzyme activity, CYP3A4 mRNA expression and CYP3A4 protein expression in human hepatoma HepG2 and colon adenocarcinoma LS174T cells: (**A**) CYP3A4 enzyme activity in HepG2 cells after incubation for 48 h; (**B**) CYP3A4 mRNA expression in HepG2 cells and LS174T cells after incubation for 24 h; (**C**) CYP3A4 protein expression in HepG2 cells after incubation for 24 h; (**D**) CYP3A4 protein expression in HepG2 cells after treatment with fucoxanthin in combination with rifampin. Values are means ± SD, *n* = 3; means without a common letter differ significantly (*p* < 0.05).

To assess the effect of fucoxanthin on the basal and rifampin-induced CYP3A4 enzyme activity, HepG2 cells were treated with fucoxanthin (1–10 μM) alone or in the combination with human PXR (hPXR) inducer (20 μM rifampin) for 48 h. As shown in Figure 1A, treatment of HepG2 cells with fucoxanthin (1–10 μM) for 48 h significantly decreased the basal CYP3A4 enzyme activity (21% decrease, *p* < 0.05, at 10 μM fucoxanthin), as compared with that of untreated cells. Co-incubation of cells with fucoxanthin (1–10 μM) and rifampin (20 μM) significantly attenuated rifampin-induced CYP3A4 enzyme activity, and the inhibitory effect of fucoxanthin was concentration-dependent (26% decrease, *p* < 0.05, at 10 μM fucoxanthin) (Figure 1A).

### 2.2. Fucoxanthin Inhibits the Basal and Attenuated Rifampin-Induced CYP3A4 mRNA Expression in HepG2 and LS174T Cells

To elucidate whether the decreased CYP3A4 enzyme activity induced by fucoxanthin was due to the decreased mRNA expression, we used reverse transcriptase real-time PCR for CYP3A4 mRNA assessment. We found that fucoxanthin (1–10 μM) significantly decreased the basal CYP3A4 mRNA expression in HepG2 and LS174T cells after incubation for 24 h (39%, *p* < 0.05 and 78%, *p* < 0.001, respectively, at 10 μM fucoxanthin), as compared with untreated cells (Figure 1B). Fucoxanthin (1–10 μM) also significantly decreased rifampin-induced CYP3A4 mRNA expression in HepG2 cells and LS174T cells, with a 53% (*p* < 0.001) and a 65% (*p* < 0.001) inhibition, respectively, after incubation with 10 μM fucoxanthin for 24 h, as compared with rifampin-treated cells (Figure 1B).

### 2.3. Fucoxanthin Inhibits the Basal and Attenuated Rifampin-Induced CYP3A4 Protein Expression in HepG2 Cells

Western blotting was performed to evaluate the protein levels of CYP3A4. We found that fucoxanthin (1–10 μM) significantly decreased the basal CYP3A4 protein expression in a concentration-dependent manner (33%, *p* < 0.05, at 10 μM fucoxanthin, as compared with solvent control) (Figure 1C). Co-incubation of cells with fucoxanthin (1–10 μM) and rifampin (20 μM) significantly decreased rifampin-induced CYP3A4 protein expression (to the level of untreated cells), although the effect was not concentration-dependent (Figure 1D). These results are consistent with those of mRNA expression.

### 2.4. Fucoxanthin Inhibits PXR-Mediated CYP3A4 Promoter Activity in HepG2 Cells

**Figure 2:**
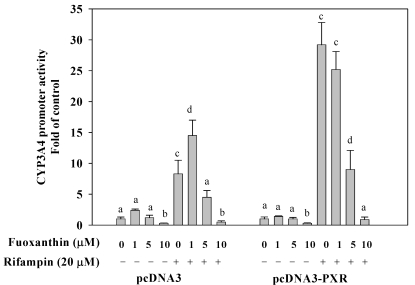
Effects of fucoxanthin (0–10 μM) alone or fucoxanthin plus rifampin (20 μM) on human PXR-CYP3A4 promoter expression in HepG2 cells after incubation for 24 h. Values are means ± SD, *n* = 3; means without a common letter differ significantly (*p* < 0.05).

Since hPXR is a dominant regulator of CYP3A4 expression, we assessed the inhibition of fucoxanthin on rifampin-induced hPXR transactivation activity on CYP3A4 promoter. As shown in Figure 2, 10 μM fucoxanthin significantly decreased the basal CYP3A4 promoter activity (70% decrease, as compared with the untreated group, *p* < 0.001). Treatment of HepG2 cells with fucoxanthin (1–10 μM) for 24 h also significantly attenuated the activation of PXR-mediated CYP3A4 promoter induced by rifampin, and the effect of fucoxanthin was concentration-dependent, with 10 μM producing the greatest inhibitory effect (97% decrease, as compared with rifampin treatment alone, *p* < 0.001).

### 2.5. Fucoxanthin Inhibits the Basal and Attenuated Rifampin-Induced MDR1 mRNA Expression in HepG2 and LS174T Cells

**Figure 3:**
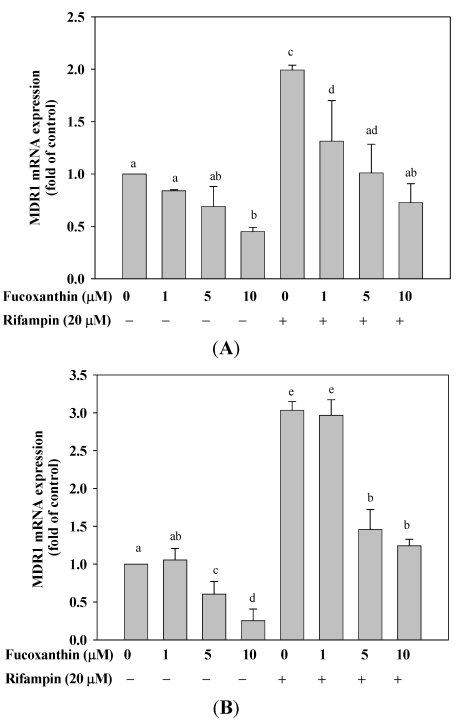
Effects of fucoxanthin (0–10 μM) on themultiple drug resistance 1 (MDR1) mRNA expression in HepG2 cells and LS174T cells after incubation for 24 h. Total DNA was isolated and the expression of indicated gene was determined by real-time PCR. (**A**) HepG2 cells; (**B**) LS174T cells. Values are means ± SD, *n* = 3; means without a common letter differ significantly (*p* < 0.05).

We also evaluated the effect of fucoxanthin on mRNA expression of MDR1, another PXR-regulated gene. We found that fucoxanthin (1–10 μM) significantly decreased the basal expression of MDR1 mRNA in HepG2 (Figure 3A) and LS174T (Figure 3B) cells after incubation for 24 h (55%, *p* < 0.01 and 59%, *p* < 0.001, respectively, at 10 μM fucoxanthin, as compared with the untreated group). Moreover, the induction of MDR1 mRNA expression by rifampin (20 μM) was attenuated by co-incubation with fucoxanthin (1–10 μM) for 24 h in both HepG2 and LS174T cells, with a 53% (*p* < 0.01) and a 63% (*p* < 0.001) inhibition, respectively, at 10 μM fucoxanthin, as compared with rifampin treatment alone. 

### 2.6. Fucoxanthin Inhibits hCAR- and rPXR-Mediated CYP3A4 Promoter Transactivation in HepG2 Cells

**Figure 4:**
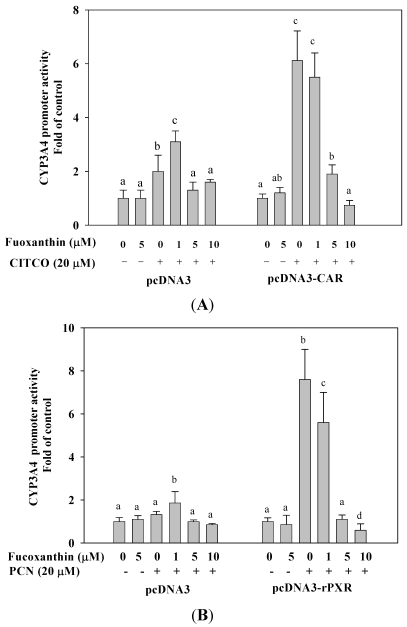
Effects of fucoxanthin (0–10 μM) alone or fucoxanthin plus rifampin (20 μM) on human constitutive androstane receptor (CAR) and mouse PXR-mediated CYP3A4 promoter expression in HepG2 cells after incubation for 24 h. (**A**) human CAR-mediated CYP3A4 promoter expression; (**B**) mouse PXR-mediated CYP3A4 promoter expression. Values are means ± SD, *n* = 3; means without a common letter differ significantly (*p* < 0.05).

The CYP3A4 expression is also regulated by human constitutive androstane receptor (hCAR). Similar reporter assays were performed in HepG2 cells to evaluate the effect of fucoxanthin on hCAR-mediated CYP3A4 transactivation. We found that fucoxanthin (1–10 μM) markedly attenuated [6-(4-chlorophenyl)imidazo[2,1-*b*][1,3]thiazole-5-carbaldehyde-*O*-3,4-dichlorobenzyl) oxime], (CITCO)-induced CYP3A4 promoter activation through CAR (88%, *p* < 0.001, as compared with CITCO treatment alone, at 10 μM fucoxanthin) (Figure 4A). In addition, we found that fucoxanthin strongly attenuated rat-PXR mediated CYP3A4 promoter activity induced by 5-pregnen-3β-ol-20-one-16α-carbonitrile (PCN), whereas the effect of fucoxanthin at 10 μM was lower than for untreated cells (Figure 4B). The results indicate that fucoxanthin does not have a species-specific effect on human and rat PXR. In control experiments without overexpression of nuclear receptor, neither PXR nor CAR activity was increased or inhibited by fucoxanthin treatment. 

### 2.7. Fucoxanthin Disrupt the Interaction of PXR and Coactivator Interactions in HepG2 Cells

**Figure 5:**
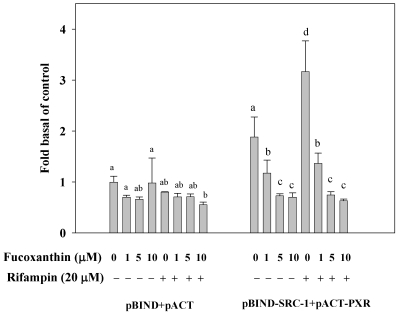
Effects of fucoxanthin (0–10 μM) alone or fucoxanthin plus rifampin (20 μM) on PXR co-activator interactions in HepG2 cells after incubation for 24 h. Cells were transfected with a pBIND-SRC-1 and pCAT-PXR expression vector. Values are means ± SD, *n* = 3; means without a common letter differ significantly (*p* < 0.05).

We then evaluated that the effect of fucoxanthin on the interaction between PXR and coactivator (SRC-1) in HepG2 cells, as determined by the mammalian two-hybrid assay. As shown in Figure 5, fucoxanthin (1–10 μM) significantly decreased the interaction between PXR and SRC-1 in HepG2 cells after incubation for 24 h. In contrast, rifampin strongly promoted the specific interaction of PXR and SRC-1, whereas fucoxanthin significantly and concentration-dependently, attenuated rifampin-induced interaction between PXR and SRC-1, with an 80% inhibition (*p* < 0.001) at 10 μM fucoxanthin. The results indicate that fucoxanthin disrupts the interaction between PXR and SRC-1 both on the basal and rifampin-induced interaction, thereby potentially preventing transcriptional activation of PXR-regulated genes.

## 3. Discussion

The main questions addressed by this study were whether fucoxanthin overcomes drug resistance and whether the effect of fucoxanthin is associated with attenuation rifampin-induced CYP3A4 and MDR1 gene expression by PXR- mediated pathways in HepG2 hepatoma cells. We demonstrate here for the first time that fucoxanthin exhibits anti-drug resistance potential and that this effect is likely to be associated with inhibition of PXR-regulated genes, including CYP3A4 and MDR1.

The full activity of nuclear receptors (NRs) depends on a large number of cellular factors that do not bind DNA directly but are selectively recruited to the promoter by NRs through protein-protein interaction. A probable mechanism by which fucoxanthin attenuates rifampin-induced CYP3A4 and MDR1 is through inhibition of the interaction between PXR and SRC-1, a coactivator of PXR. Several coactivators have been shown to be important for nuclear receptor activation, including the SRC-1, transcriptional intermediary factor (TIF) 2, the activator of thyroid and retinoic acid receptor (ACTR), and peroxisome proliferator-activated receptor-binding protein (PBP) [31]. The binding of nuclear receptors to their ligands causes a conformational change, resulting in dissociation of corepressor proteins and recruitment of coactivator proteins, thereby potentially activating transcriptional levels of nuclear receptor-regulated genes [32]. In the present study, we found that fucoxanthin (1–10 μM) significantly and concentration-dependently attenuated rifampin-induced interaction between PXR and SRC-1, suggesting that fucoxanthin may inhibit PXR coactivator recruitment potential, thereby potentially preventing ligand-mediated PXR transcriptional activation of PXR-regulated genes. In the human CYP3A4 promoter, PXR can act as a heterodimer with RXRα, which then binds to an everted repeat, separated by 6 bp (ER6), located in the proximal promoter and a three-nucleotide spacer (DR3) as well as ER6 in the distal xenobiotic responsive enhancer module (XREM). This is located approximately 8 kbs upstream of the transcription start site [33,34]. We show here that fucoxanthin significantly attenuated rifampin-induced CYP3A4 and MDR1 expression through reduced PXR-mediated CYP3A4 promoter activity. 

The induction of both CYP450 and MDR1 genes at the transcriptional level is generally mediated by a PXR, CAR, glucocorticoid receptor (GR), and a vitamin D receptor (VDR) [35]. Among these receptors, PXR and CAR are members of the same nuclear receptor subfamily (NR1), sharing ~40% amino acid identity in their ligand binding domains (LBDs), which regulate an overlapping but not identical set of genes [36]. In the present study, we found that fucoxanthin (1–10 μM) strongly attenuated CAR-mediated CYP3A4 promoter activity induced by CITCO. Moreover, previous reports revealed that PXR and CAR exhibit promiscuous, low-affinity ligand binding characteristics [37]. Hence, CAR and PXR appear to share a similar mechanism, by recruiting SRC-1 in CYP3A4 regulation [38]. Thus, PXR and CAR are considered the key transcriptional regulators of CYP3A4 and MDR1 [39].

PXR activation has been implicated in a number of clinically significant adverse drug–drug interactions and in increased cancer cell malignant phenotypes in response to PXR activation by drugs [20]. Hence, development of oral PXR antagonists with rapid absorption within the stomach and high hepatic extraction may be beneficial in preventing undesirable side effects in chronic patients who must take medications that are PXR activators. A lot of evidence exists to show that many PXR antagonists have been identified *in vitro*, such as the antineoplastic agent ecteinascidin-743 (ET-743), a marine-derived compound from the ascidian Ecetinascidia turbinanta [31], the antifungal agent ketoconazole (and related azoles) [40], the human HIV protease inhibitor, A-792611 [41], the phytoestrogen coumestrol [42], a topisomerase I inhibitor camptothecin [43], metformin [44] and the dietary isothiocyanate sulforaphane [45]. These are all functional inhibitors of PXR. In the present study, we found that fucoxanthin strongly attenuated rifampin-induced PXR activation, suggesting that fucoxanthin may be a PXR antagonist.

## 4. Experimental Section

### 4.1. Chemicals and Reagents

Dulbecco’s modified eagle medium (DMEM), fetal bovine serum (FBS), trypsin, penicillin, sodium pyruvate, and non-essential amino acids (NEAA) were purchased from GIBCO/BRL (Maryland, USA). Rifampin, 5-Pregnen-3β-ol-20-one-16α-carbonitrile (PCN), 6-(4-Chlorophenyl) imidazo[2,1-*b*][1,3] thiazole-5-carbaldehyde *O*-(3,4-dichlorobenzyl) oxime (CITCO) were from Sigma-Aldrich (St. Louis, Missouri, USA) and dissolved in DMSO at appropriate concentrations. Anti-CYP3A4 goat polyclonal antibody and secondary antibody were from Santa Cruz (Santa Cruz, CA, USA). Fucoxanthin was extracted from *Undaria pinnatifida* and purified, as we reported previously [46]. The purity of fucoxanthin obtained as described above was 99.2%. The purified fucoxanthin was dissolved in ethanol to a final concentration of 10 mM as the stock solution. Before the experiment, fucoxanthin solutions were prepared freshly in a mixture of ethanol and FBS (1:9), as adopted from the preparation of lycopene solution [47]. 

### 4.2. Cell Culture

The human hepatoblastoma cell line HepG2 and human colon adenocarcinoma cell line LS174T were obtained from Food Industry Research and Development Institute (FIRDI, Taiwan) and maintained in DMEM supplemented with 10% fetal bovine serum without antibiotics, under 5% CO_2_ at 37 °C. 

### 4.3. Plasmids Construction

Plasmids pcDNA3-hPXR and pGL3B-CYP3A4 [(−444/+53) (−7836/−7208)] containing the full-length human PXR and CYP3A4 promoter reporter construct, respectively, have been reported previously [48]. A fragment encoding residue 595–800 of human SRC (GenBank accession number U90661) and full-length PXR was cloned into the pBIND-GAL4 and pACT-VP16 vector to become pBIND-SRC-1 and pACT-PXR, respectively, as described earlier [49]. A full-length human CAR cDNA (GenBank accession number NM_001077480) was purchased from Open Biosystems (Odyssey Drive Huntsville, Alabama, USA) and a full-length rat PXR (GenBank accession number NM_052980) was cloned from cDNA of rat liver. Both PCR products were amplified from cDNA using primers (for human CAR: forward primer, 5′-AAG GAT CCA CGT CAT GGC CAG TAG-3′; reverse primer, 5′-CCA ATC TAG AGC ATT TTC CCA CTC-3′; for rat PXR: forward primer, 5′-GAT GGG ATC CTG GAG ATG AGA CCT GAG G-3′; reverse primer, 5′-CTC ATC TAG AGC CAC TCA GCC GTC CGT G-3′). The PCR product was digested with *Bam*HI and *Xba*I, and restriction enzyme cut sites were introduced into the primers before the PCR. The cut fragment was cloned into the pcDNA3 vector with corresponding restriction enzyme sites to generate pcDNA3-hCAR and pcDNA3-rPXR, respectively. The reporter construct, pG5luc and internal control plasmid pRC-CMV-β-galactosidase, were purchased directly from Promega and Invitrogen (Groningen, The Netherlands), respectively.

### 4.4. Determination of CYP3A4 Enzymatic Activities Using P450-Glo™ Assay

CYP3A4 enzyme activity was measured using P450-Glo™ assays (Promega, USA). Briefly, cells (3 × 10^5^/mL) were subcultured into 12-well plates and were incubated with various concentrations of fucoxanthin. After incubation for 48 h, the medium was discarded, and the cells were rinsed twice using phosphate buffer saline. Fresh medium containing 50 μM luciferin-PFBE was added to cells and incubated for 3 h at 37 °C. Subsequently, 50 μL of the medium from each well was transferred to a 96-well opaque white luminometer plate, and 50 μL of luciferin detection reagent was added to initiate a luminescent reaction and then stabilized for 20 min. Luminescence was read using a luminometer, and luminescence signals were calculated by subtracting background luminescence values (no-cell control) from the values of the test compound and the blank (without the test compound).

### 4.5. Real-Time Polymerase Chain Reaction of CYP3A4 and MDR1

Total RNA in cell cultures was extracted with REzol reagent (PROtech Technologies, Inc.), and 1 μg of total RNA was reverse-transcribed by using oligo-dT as a primer in 20 μL reverse-transcription solutions containing 1 μL reverse transcriptase (Promega, USA). Real-time PCR was performed with a Corbett instrument (Applied Biosystems) using SYBR Green Master Mix (ProTech, USA) according to the manufacturer’s instructions. In all real-time PCR experiments both a non-template control (NTC) and a standard curve were amplified as well. The RNA abundance was normalized to β-actin RNA in each sample. The primers and PCR condition used in this study are shown in Table 1.

**Table 1:** Primer sequences used for real-time polymerase chain reaction.

Gene	5′-3′ sequence	PCR condition (45 cycles)
*CYP3A4*		
Forward	5′-GGGAAGCAGAGACAGGCAAG-3′	Denaturation
Reverse	5′-GAGCGTTTCATTCACCACCA-3′	(30 s at 95 °C)
*MDR1*		
Forward	5′-AAAAAGATCAACTCGTAGGAGTA-3′	Annealing
Reverse	5′-GCACAAAATACACCAACAA-3′	(30 s at 60 °C)
*β-actin*		
Forward	5′-GTGGGGCGCCCCAGGCACCA-3′	Extension
Reverse	5′-CACCCCGCGGGGTCCGTGGT-3′	(30 s at 72 °C)

### 4.6. Western Blotting

Protein expression of CYP3A4 was measured by western blotting. In cell culture experiments, the medium was removed and cells were rinsed with phosphate buffered saline (PBS) twice. After the addition of 0.5 mL of cold radioimmunoprecipitation assay (RIPA) buffer and protease inhibitors, the cell lysates were then subjected to a centrifugation of 10,000 g for 30 min at 4 °C. Total protein (50 μg) from the supernatant was resolved on SDS-PAGE and transferred onto a polyvinylidene fluoride (PVDF) membrane. After blocking with Tris-buffered saline (TBS) buffer (20 mmol/L Tris-HCl, 150 mmol/L NaCl, pH 7.4) containing 5% nonfat milk, the membrane was incubated with monoclonal antibody against human CYP3A4 followed by incubation with horseradish peroxidase-conjugated anti-goat IgG, and then visualized using an ECL chemiluminescent detection kit (Amersham, Sweden). The relative density of the immunoreactive bands was quantitated by densitometry (Gel Pro Analyzer TM, version 3.0, Media Cybernetics, USA).

### 4.7. Transient Transfection, CYP3A4 Reporter Assay, and Mammalian Two-Hybrid Assay

HepG2 cells (1.8 × 10^4^ cells/well) were plated in 96-microwell-white-plates (Nalge Nunc, Rochester, New York, USA) before transfection. For CYP3A4 reporter assay, plasmid DNA is introduced using the PolyJET™ (SignaGen Laboratories, Ijamsville, MD), whereas, for mammalian two-hybrid assays, we used Effectene^®^ Transfection Reagent (Qiagen, Valencia, California, USA) according to the manufacturer instructions. For CYP3A4 reporter assays, 0.15 µg of CYP3A4 reporter construct, 0.02 µg of control β-galactosidase plasmid, and each 5 ng of pcDNA 3 vector or PXR expression plasmid were added per well, and the mixture was allowed to stand for 6–7 h. The cells were then exposed to rifampin/CITCO/PCN and/or fucoxanthin for 24 h before being lysed *in situ* with 80 µL of Glo Lysis Buffer (Promega, Madison, Wisconsin, USA). Forty microliters of clear lysates were used for β-galactosidase assay. A 40-µL aliquot of each clear lysate was used for reporter assay, after which 40 µL ONE-Glo™ Luciferase Assay System (Promega, Madison, Wisconsin, USA) was added to the lysates. For mammalian two-hybrid assays, 0.1 µg of pG5luc reporter gene, 0.04 µg each of pBIND-GAL4 and pACT-VP16 constructs, and 0.02 µg of control β-galactosidase plasmid were added per well. After incubation for 6–7 h, the cells were exposed to rifampin and/or fucoxanthin, and after a further 24 h, the cells were lysed *in situ* with 90 µL of Cell Culture Lysis Reagent (Promega, USA). A 40-µL aliquot of each clear lysate was used for β-galactosidase assay, after which 80 µL Luciferase Assay System (Promega) was applied into the residual lysates. Luminescent signal was measured by using a luminescence multi-mode microplate reader (Synergy HT, BioTek Instruments Inc., Vermont, USA). Luciferase activities were normalized by the corresponding β-galactosidase activity.

### 4.8. Statistical Analysis

All experiments were repeated at least three times. Values are expressed as means ± SD and analyzed using one way ANOVA followed by LSD test for comparisons of group means. All statistical analyses were performed using SPSS for Windows, version 10 (SPSS, Inc.); a *p* value < 0.05 is considered statistically significant.

## 5. Conclusions

In conclusion, the present study demonstrates that fucoxanthin exhibits anti-drug-resistance potential and that the effect is likely to be associated with attenuated interaction between PXR and SRC-1, thereby potentially preventing activation of PXR-mediated CYP3A4 promoter expression. This leads to inhibition of PXR-regulated gene expression, including CYP3A4 and MDR1. The results suggest the potential use of fucoxanthin as an adjuvant to prevent drug-resistance in patients receiving chronic therapy with PXR agonists. 
